# Delineating intra-tumoral heterogeneity and tumor evolution in breast cancer using precision-based approaches

**DOI:** 10.3389/fgene.2023.1087432

**Published:** 2023-08-17

**Authors:** Kutlwano Rekgopetswe Xulu, Ekene Emmanuel Nweke, Tanya Nadine Augustine

**Affiliations:** ^1^ School of Anatomical Sciences, Faculty of Health Sciences, University of the Witwatersrand, Johannesburg, South Africa; ^2^ Department of Surgery, School of Clinical Medicine, Faculty of Health Sciences, University of the Witwatersrand, Johannesburg, South Africa

**Keywords:** breast cancer, breast cancer genetics, signaling pathways, precision medicine, targeted therapy, tumor evolution, tumor heterogeneity

## Abstract

The burden of breast cancer continues to increase worldwide as it remains the most diagnosed tumor in females and the second leading cause of cancer-related deaths. Breast cancer is a heterogeneous disease characterized by different subtypes which are driven by aberrations in key genes such as *BRCA1* and *BRCA2*, and hormone receptors. However, even within each subtype, heterogeneity that is driven by underlying evolutionary mechanisms is suggested to underlie poor response to therapy, variance in disease progression, recurrence, and relapse. Intratumoral heterogeneity highlights that the evolvability of tumor cells depends on interactions with cells of the tumor microenvironment. The complexity of the tumor microenvironment is being unraveled by recent advances in screening technologies such as high throughput sequencing; however, there remain challenges that impede the practical use of these approaches, considering the underlying biology of the tumor microenvironment and the impact of selective pressures on the evolvability of tumor cells. In this review, we will highlight the advances made thus far in defining the molecular heterogeneity in breast cancer and the implications thereof in diagnosis, the design and application of targeted therapies for improved clinical outcomes. We describe the different precision-based approaches to diagnosis and treatment and their prospects. We further propose that effective cancer diagnosis and treatment are dependent on unpacking the tumor microenvironment and its role in driving intratumoral heterogeneity. Underwriting such heterogeneity are Darwinian concepts of natural selection that we suggest need to be taken into account to ensure evolutionarily informed therapeutic decisions.

## Introduction

Breast cancer is a disease marked by considerable heterogeneity. Challenges in accurate diagnosis and effective therapy, even within clinically defined subtypes, is not only affected by intratumoral heterogeneity but by the interplay between cell types in the tumor microenvironment (TME) that drives tumor progression and shapes the response to therapy. Genomic instability is suggested to occur during early-stage neoplastic transformation where it may contribute to setting the stage for evolution (Tarabichi et al., 2013). Within the TME, tumors generate clones under selective pressures that enhance their capacity to thrive and change their ecological niche in the TME. These ecological niches are also spatiotemporally defined, permitting clones to adapt the TME landscape to enable invasion and metastasis as they acquire more aggressive phenotypes (Bechtel, 2019; Bukkuri et al., 2023). This may reflect the accumulation of gradual microevolutionary changes or larger shifts that account for macroevolutionary changes, which while controversial in their mechanism (Gerlinger et al., 2014), nevertheless result in intratumoral heterogeneity that highlights the evolvability and adaptability of tumors. This potentially accounts for poor response to treatment, resistance and recurrence (Gallaher et al., 2019; Lei, 2020; Boddy, 2022; Bukkuri et al., 2023). In this review, we will first revisit breast tumor subtypes that describe intertumoral heterogeneity and are affected by spatiotemporal dynamics that underlie acquisition of more aggressive phenotypes. We will then uncover the tumor microenvironment while considering the concept of intratumoral heterogeneity as a function of tumor evolution. In identifying the challenges associated with breast cancer diagnosis and the underlying impact of genomic heterogeneity, we will present advancements in technologies for precision diagnoses, and suggest the need for evolutionarily informed therapeutic decisions.

## Breast cancer molecular subtypes and associated alterations

The burden of breast cancer continues to increase worldwide. Of an estimated 19.3 million newly diagnosed cancer cases, breast cancer in females remains the most commonly diagnosed cancer and a leading cause of cancer-related deaths (Sung et al., 2021). Studies that have reported a reduction in breast cancer-related mortality over 20 years have attributed this to improved screening resulting in early diagnosis, and better treatment strategies for localized and metastatic disease (Siegel et al., 2015; Sundquist et al., 2017). Breast cancer incidence in low- and middle-income countries (29.7 per 100,000) remains lower than that identified in high-income countries (55.9–>80 per 100,000). The high incidence of breast cancer in high-income countries has been associated with hormonal risk factors including early age at menarche and later age at menopause; an advanced age at the birth of the first child, fewer children and less breastfeeding (Sung et al., 2021), compared to African patients from sub-Saharan Africa (Brandão et al., 2021). In high-income countries, oral contraceptive use and hormone replacement therapy, as well as lifestyle risk factors, have also been implicated in the rising incidence of breast cancer (Sung et al., 2021). Data from developed countries including the US, Denmark, Ireland and Scotland point to a rise in estrogen-dependent tumors with a concurrent reduction in estrogen-independent tumors. This is postulated to accompany the global obesity issue and also be a result of more sophisticated mammographic screening better able to detect slow-growing tumors (Sung et al., 2021). Comparatively, poor resources in low- and middle-income countries impact access to mammographic screening, diagnosis and treatment plans (Galukande et al., 2014). As such, despite a lower incidence of breast cancer in low- and middle-income countries, there remains a markedly higher mortality rate (17%), with patients typically presenting with late-stage tumors (Sung et al., 2021). Moreover, the distribution of subtypes is difficult to unravel in sub-Saharan Africa, since profiling of hormone-receptor status is not routine (Galukande et al., 2014; Sengal et al., 2017), except in some of the Southern African countries with better healthcare resources including Namibia and South Africa (Hercules et al., 2022). Despite this limitation, studies attempting to understand the immunohistochemical landscape of breast cancer illustrate that a larger proportion of cases present at a more advanced stage in younger, pre-menopausal patients (Jedy-Agba et al., 2016; Azubuike et al., 2018). Although estrogen receptor-positive (ER+) breast cancer remains a dominant phenotype in Africa (Kakudji et al., 2021; Popli et al., 2021), a substantive increase in other subtypes, particularly triple-negative breast cancer or basal-like tumors in Uganda (Galukande et al., 2014), Eritrea and Sudan (Sengal et al., 2017), has been found compared to developed countries (Sung et al., 2021; Hercules et al., 2022). Moreover, triple-negative breast cancer frequency is high in Caribbean and North American populations with West African ancestry, indicating a heritable factor that has yet to be fully delineated (Hercules et al., 2022).

Histologically breast cancer is classified into three main groups that reflect the bulk of intertumoral heterogeneity: estrogen-dependent, HER2 over-expressing and triple-negative breast cancer (TNBC) (Hamdan et al., 2019). Over time an important distinction has been made to further characterize the histological classification and use the molecular profile of the tumor to explain the properties of the tumor, and to an extent, the vast genetic heterogeneity. Over the years various molecular subtypes for breast cancer have been defined as Luminal A, Luminal B, HER2-enriched, the basal-like and normal-like (Sørlie et al., 2001), which reflect to a large extent, immunohistochemistry-derived clinical categories (Belizario and Loggulo, 2019). The classification of these tumors into various molecular subtypes is not only reflected through immunohistochemical classification of hormone receptor status but can also be characterized at the genomic and transcriptomic level. These molecular subtypes of breast cancer consist of a unique profile that may better describe the underlying biology of these tumors (Tishchenko et al., 2016).

## Luminal subtypes

Hormone-receptor-positive tumors constitute up to 80% of breast cancer cases (Turashvili and Brogi, 2017). Luminal A and B tumors phenotypically present with high ER expression, that reflects underlying molecular subtype classification. For example, when gene expression in luminal A and luminal B tumors were compared with the designated PAM50 gene set, differences were observed in genes involved in the cell cycle and cell proliferation such as *BIRC5*, *CCNB1*, *CDC20*, *CEP55*, *KIF2C*, MELK, MKI67 and UBE2C (Tishchenko et al., 2016). Pathway enrichment shows that luminal A tumors amplify pathways associated with extracellular matrix organization and collagen formation, comtrasting to luminal B tumors that show enrichment of DNA repair pathways, similar to that of HER2-enriched tumors (Wang et al., 2022). Phenotypically, luminal A tumors expressing ER and PR have the lowest proliferation potential (Ki-67), and present with the best prognosis (Goldhirsch et al., 2013). Luminal B tumors express comparatively lower levels of ER and PR, but have higher proliferative potential and are more aggressive (Goldhirsch et al., 2013). While immunohistochemical cut-offs as low as 1% for ER/PR positivity have been suggested to be responsive to hormone therapy, the latest American Society of Clinical Oncology/College of American Pathologists (ASCO/CAP) guidelines recommend a new category, ER low positive, for samples that have between 1% and 10% ER positivity (Allison et al., 2023). Such patients may not benefit as much from hormone therapy due to a reduction in ER-associated gene signatures implicated in tumor progression (Grant et al., 2019; Allison et al., 2023). Typical hormone therapies include selective estrogen receptor modulators such as tamoxifen, selective estrogen receptor degraders such as fulvestrant, widely used for both pre- and post-menopausal patients; and aromatase inhibitors such as letrozole and anastrozole, also used in post-menopausal patients (Palmieri et al., 2014; Janku et al., 2018; Belizario and Loggulo, 2019). Variations in response to therapy can also be described by intratumoral heterogeneity concerning the expression of these hormone receptors (Iwamoto et al., 2016; Turashvili and Brogi, 2017). Moreover, there remains variability in the diagnostic accuracy of immunohistochemical reporting, dependent on tissue acquisition, storage and other technical factors, all of which may impact prognosis (Turashvili and Brogi, 2017; Grant et al., 2019).

Mutational profiles that result in reduced functional activity, for example, splice variants in the ER, may lead to a positive immunohistochemical detection, but poor response to hormone therapy (Groenendijk et al., 2013; Grant et al., 2019), as such molecular profiling for specific targets is indicated. *ESR1* mutations, particularly of residues associated with the ligand binding domain, while rare in primary breast cancer, have an increased frequency in metastatic or recurrent disease. Such mutational acquisitions like *ESR1*, are clonally selected for depending on surrounding selective pressures (Ren et al., 2021) and can permit constitutive activation of the ER and resistance to treatment (Brett et al., 2021). In addition to *ESR1* mutations (loss, amplification and translocation), endocrine resistance can also be mediated by changes in pathways including PI3K-AKT-mTORC1, RAS-MAPK and CDK4/6-RB-E2F (Brett et al., 2021). The luminal A phenotype is also associated with mutations in phosphatidylinositol-3-kinase (*PIK3CA*), mitogen-activated protein kinase (*MAP3K1*), GATA binding factor and *TP53* (Belizario and Loggulo, 2019), some of the most commonly detected mutations (Figure 2). Luminal A tumors are also more likely to retain *Rb1* gene signatures (The Cancer Genome Atlas Network, 2013). The more aggressive luminal B tumors and HER2-over-expressing tumors typically harbor *TP53* mutations (Belizario and Loggulo, 2019). In metastatic breast cancers, the mutational landscape is even greater than in primary tumors, evident in driver genes including *TP53*, *AKT1*, *ESR1*, *GATA3, NF1*; moreover, greater clonal diversity is evident (Bertucci et al., 2019), reflecting greater complexity within the TME. Notably, a recent study using computational multiplex mapping illustrated that the TME network is a better predictor of *TP53* mutations than tumor cell phenotype alone, revealing dynamic reciprocity between the cell types within the TME (Danenberg et al., 2022). Genomic heterogeneity illustrated by DNA copy number profiling also highlights the variability between luminal tumors; luminal A tumors typically classified as lower grade, have a gain of function in chromosome 1 (1q) and a loss of function in chromosome 16 (16q). Thre more aggressive luminal B tumors are associated with amplification in chromosomes 8p11 (FGFR1 locus), 8q21,11q13, 20q13 and 17q12 (HER2 locus), with the latter leading to HER2-amplified luminal phenotype (Tishchenko et al., 2016).

## HER2 enriched subtypes

The HER2-enriched subtype, accounting for approximately 15%–20% of breast cancer is characterized by high expression of the *ERBB2* gene, but shows considerable heterogeneity in its presentation which may also include differences in ER+ expression (ER+/ER-) (Turashvili and Brogi, 2017). HER2 protein expression scored using immunohistochemistry (IHC) include: 3+ (complete membrane expression), 2+ (weak to moderate membrane expression), 1+ (no expression) (Popović et al., 2023). HER2 positive tumors are defined by IHC score of 2+ and amplification of *ERBB2* gene. In contrast, HER2-tumors (1+) may have a IHC score of 2+ not associated with *ERBB2* gene amplification or no HER2 expression (0) (Popović et al., 2023). The HER2-enriched/ER-subtype has also been defined as a molecular apocrine phenotype, expressing elevated levels of androgen receptor target genes (Turashvili and Brogi, 2017). HER2 phosphorylation leads to constitutive activation of signaling pathways, including the PI3K and MAPK pathways involved in cell survival, proliferation, and angiogenesis (Fujimoto et al., 2020); moreover, HER2-enriched tumors also typically show *FGFR2* mutations and amplifications *KRAS* (Belizario and Loggulo, 2019). Notably, some tumors show *ERBB2* gene amplification without HER2 protein expression, which has repercussions for therapeutic response (Turashvili and Brogi, 2017). HER2-low tumors, although difficult to detect accurately, are proving increasingly to be a variation of breast cancer that while showing no benefit from traditional HER2-based therapies, including Trastuzumab and/or lapatinib (Belizario and Loggulo, 2019), are responsive to antibody-drug conjugates (ADC) including Trastuzumab-deruxtecan (T-Dxd) (Baez-Navarro et al., 2022). Mutations in *PIK3CA*, common in approximately 25% of breast cancers, particularly in HER2-enriched tumors, also confer resistance to targeted HER2-based therapies (Belizario and Loggulo, 2019). Such findings beg the question of the existence of tumor clones with intrinsic resistance, compared to the acquired resistance that tumor cells may evolve in the TME.

## Triple-negative breast cancer (TNBC) subtypes

The TNBC subtype is characterized with IHC, by a lack of (or low expression of) ER and PR (≤1%), and HER2 (between 0 and 1+) according to the ASCO/CAP guidelines, with genomic and transcriptomic studies additionally providing greater evidence of its heterogeneity (Burstein et al., 2015; Almansour, 2022). TNBC is thus ineligible for hormone therapies or HER2-targeting therapies but is rather managed with chemotherapy, including taxanes and anthracycline (Kim et al., 2018). TNBC is regarded as the most aggressive tumor subtype due to genomic instability, and alterations in *TP53* and DNA-repair genes such as *BRCA1*, remaining highly variable in its molecular profile (Perou, 2010). The TNBC subtype displays heterogeneity depending on *TP53* status (Lehmann et al., 2011) with alterations in *PIK3CA*, *MYC* and *PTEN* dominating the landscape (Millis et al., 2015). Lehmann and others in their seminal study identified 6 subtypes of TNBC: androgen-receptor positive, claudin-low mesenchymal, mesenchymal stem-like, immunomodulatory, basal-like 1 and basal-like 2. Despite being regarded as ER-, up to 55% of TNBC can be positive for the androgen receptor (AR) (Lehmann et al., 2011; Turashvili and Brogi, 2017), with gene ontologies showing enrichment of hormonally regulated pathways including steroid synthesis, incorporating previously described molecular apocrine subtypes (Lehmann et al., 2011). Corresponding cell line studies showed that AR + luminal subtype breast cancer cells are resistant to chemotherapy and more likely to benefit from AR antagonists (Lehmann et al., 2011). Moreover, while AR + TNBC would be histologically described as such (TNBC), due to low ER immunohistochemical detection, these tumors nevertheless present downstream activation of ER-related genes including *PGR, FOXA, GATA2* and thus may indeed respond to hormone therapy at optimal concentrations yet to be determined (Burstein et al., 2015).

TNBCs with altered *TP53* present with subtypes which respond differently to therapy; Basal-like 1 was responsive to chemotherapy, and Basal-like 2 upregulated the EGFR pathway and was resistant to chemotherapy (Lehmann et al., 2011). While these findings give important clinical information; these subtypes are not as readily distinguishable using other publicly available databases (Burstein et al., 2015). In dissecting the subtypes of TNBC further, in addition to Lehmann’s defined immunomodulatory subtype enriched in genes in the Th1/Th2, NK cell and B cell receptor pathway (Lehmann et al., 2011), Burstein and others using genomic and genetic profiling identified a basal-like immune-activated subtype and a basal-like immune-suppressed subtype, with the former associated with a better prognosis (Burstein et al., 2015). Both re-defined subtypes are independent of *TP53* mutational profile (Burstein et al., 2015), which characterizes most TNBC tumors (Millis et al., 2015). Such work has been extended by Jézéquel and others (Jézéquel et al., 2019). Cell cycle regulators are also downregulated in the TNBC mesenchymal-like subtype, which is enriched with genes facilitating cell motility, ECM-receptor interactions, and cell differentiation (Lehmann et al., 2011; Burstein et al., 2015). Lehmann also identified a mesenchymal stem-like subtype, showing a similar gene profile to that of the mesenchymal subtype, but additionally containing genes involved in various growth factor signaling pathways, angiogenesis, immune signaling, and presenting with low claudin 3, 4 and 7 (Lehmann et al., 2011). The diversity within these molecular subtypes has led to variance in descriptions of their presentation and their prognostic value. This is not restricted to the TNBC, with increasing evidence highlighting that even the luminal phenotype breast cancers may be more heterogeneous than previously thought, impacting prognosis and therapeutic response (Turashvili and Brogi, 2017).

A better understanding of the genomic landscape will enable success and improved efficacy in personalized medicines or more targeted therapies. Databases such as the NCI Genomic Data Commons (GDC) provide a view into the genomic landscape of breast cancer (Figure 2) (Grossman et al., 2016). Importantly, genomic instability is suggested to occur during early-stage neoplastic transformation where it may contribute to setting the stage for evolution (Tarabichi et al., 2013). Large-scale datasets have been developed that illustrate the intersection of genomic profiling with histologically classified breast cancers, while nevertheless illustrating the genomic heterogeneity associated with clonal evolution and adaptations that occur during treatment (Sato et al., 2016; Yates et al., 2017).

## The tumor microenvironment and its impact on intratumoral heterogeneity

**FIGURE 1:**
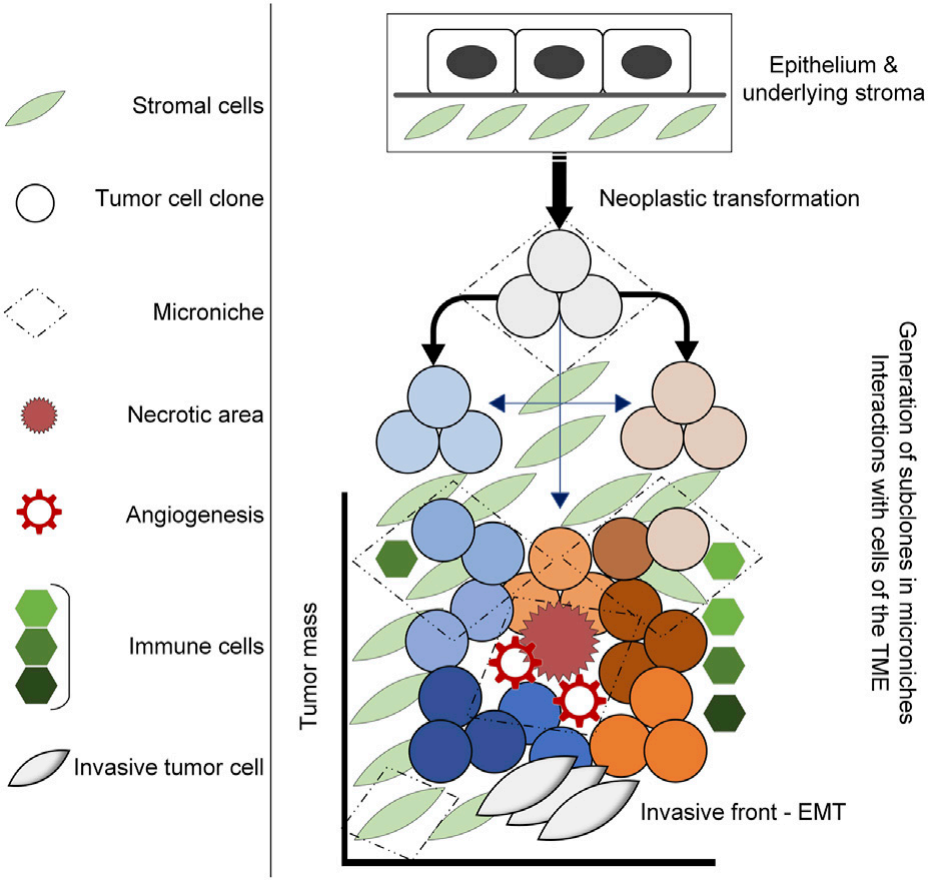
Diagram illustrating tumor development in the TME. Neoplastic transformation including the acquisition of genetic mutations, alterations in gene expression and epigenetic alterations induce the generation ot tumor clones that under selective pressures within the TME, undergo further adaptations that lead to intratumoral heterogeneity. This occurs within microniches, for example, the necrotic area that results in angiogenesis and an influx of inflammatory cells that ultimately result in adaptations that drive tumor progression.

Tumors are dynamic systems, characterized by dysregulation of proliferation, survival and growth mechanisms in transformed cells. The cancer stem cell hypothesis speaks to sustaining a pool of homogenous cells, with distinct stem-like subpopulations that drive tumor growth (Martellotto et al., 2014; Tuasha and Petros, 2020); however, there is increasing evidence that transformed cells within tumors are heterogenous, undergoing stochastic genetic and epigenetic alterations to enhance the fitness of subpopulations (Gallaher et al., 2019; Lei, 2020). Evolutionary theories of tumor growth typically focus on tumor cells themselves; however, the tumor microenvironment (TME) is being increasingly recognized for its capacity to influence tumor progression and response to therapy. As such, it is necessary to view tumors from an ecological perspective to better understand the evolutionary dynamics that drive intratumoral heterogeneity and permit tumor progression (Figure 1) (Boddy, 2022; Bukkuri et al., 2023).

Under physiological conditions, the microenvironment supports cellular function, maintains homeostasis and facilitates metabolic exchange (Sonugür and Akbulut, 2019). The composition of the TME differs significantly from that of normal tissue, in that a milieu of cells consisting of non-malignant stromal cells, infiltrating immune cell populations and other cell types are spatially and temporally organized to permit dynamic cell-cell and cell-extracellular matrix interactions. These different cells play an important role in tumor progression via their interactions with one another (Zarrilli et al., 2020). Such communication provides selective pressures that modulate expression of those genes associated with the cancer hallmarks thereby facilitating tumor evolution strategies that drive cellular plasticity and tumor heterogeneity by permitting adaptations that enhance fitness (Bechtel, 2019; Bukkuri et al., 2023). Ultimately this affects tumor cell phenotypic changes and mediates interactions with cells of the TME (Zarrilli et al., 2020; Nagy et al., 2021; Danenberg et al., 2022). Breast cancers, while not regarded as immune-hot tumors, are nevertheless infiltrated by tumor-infiltrating lymphocytes (TILs). Throughout the process of immunoediting, neoplastic cells that survive elimination by the innate or adaptive immune system, undergo rapid changes that generate genetic variants with acquired resistance to elimination that permit the establishment of a tumor (Li et al., 2021; Salemme et al., 2021; Danenberg et al., 2022). This results in a switch from immunostimulating, anti-tumor responses to immunosuppressive, pro-tumor responses, underwritten by a host of immune cell populations. For example, T regulatory cells promote an immunosuppressive TME, secreting cytokines such as IL-10 and TGF-β that prevent anti-tumor function by cytotoxic T cells and natural killer cells (Esquivel-Velázquez et al., 2015). These cytokines, also produced by regulatory B cells, are implicated in driving macrophage polarization into an M2, pro-tumorigenic phenotype (Tariq et al., 2017; Van Dalen et al., 2019), that further provides selective pressures, either secreted or via direct cell-cell contact. The results of such pressures may be genetic mutations and alterations, epigenetic modifications or biological adaptations that drive intratumoral heterogeneity by enhancing the clonal evolution of tumor cells nestled within the TME. This is accomplished by promoting tumor survival, cellular proliferation and invasion, and epithelial-mesenchymal transition (EMT) (Zarrilli et al., 2020; Leong et al., 2022; Tan and Naylor, 2022). Genes that are associated with driving these biological phenomena and thus hold clinical utility as biomarkers include: tumor survival (*PIWIL3/4*), evasion of cell death and invasion (*p53*, *COX2*, *MMP9*), EMT (*Wnt5A/B*), immune response (*PD-L1*), and growth (*Ki67*, *survivin*) (Turashvili and Brogi, 2017). Intratumoral heterogeneity will thus reflect as genetically or epigenetically distinct clones, with co-operative behaviors that manifest as enhanced survival, proliferation or migration that can be visualized histologically (Ramón y Cajal et al., 2020), for example, cells at the invasive front compared to the necrotic core (Tarabichi et al., 2013; Gallaher et al., 2019). Moreover, tumor subpopulations may also demonstrate trade-offs between biological processes, for example, between migration and proliferation (dispersal and fecundity in animal species), reflective of plasticity and phenotypic variance under the influence of spatiotemporal and constitutive TME parameters (Gallaher et al., 2019).

Tumor growth results in a central necrotic region which triggers an angiogenic switch via the production of hypoxia-inducible factors 1 (HIF-1) and HIF-2 (Sørensen and Horsman, 2020; Zarrilli et al., 2020). At oxygen levels of 2% and below, HIFs regulate downstream intracellular pathways by binding to hypoxia-responsive elements (HREs) at the enhancer and promoter regions of target genes including those involved in angiogenesis (*VEGF*), invasion (*C-MET*) and apoptosis or autophagy (*NOXA*) (Sørensen and Horsman, 2020; Li et al., 2021). At more severe hypoxia, where there is less than 0.02% oxygen available, tumor cells initiate the unfolded protein response (UPR) reacting to endoplasmic reticulum stress. This is accomplished through the PERK and BNIP3 pathways that modulate autophagic cell survival (Sørensen and Horsman, 2020). The resulting vasculature produced, by its leakiness and disorganization (Li et al., 2021), also acts as a selective pressure as different regions of a tumor may be exposed to more, or less nutritive factors, growth factors and oxygen, that in turn results in a heterogenous tumor phenotype. Ultimately, while necrosis results in the death of selected tumor cells, such microniches permit other tumor cells to adapt under stressors, thereby eliciting intratumoral heterogeneity and altering the constitution of the TME. Hypoxia regulates the influx of inflammatory cells including myeloid-derived suppressor cells, neutrophils and macrophages via the release of GM-CSF and a host of chemokines including CCL2, CCL18 and CXCL12 (Lin et al., 2015; Tariq et al., 2017). This enhances the release of reactive oxygen species (ROS), causing oxidative DNA damage in stromal and cancer cells. This has been suggested as the main factor driving genetic instability (Granger and Kvietys, 2015), thereby providing a foundation on which natural selection may act. Oxidative stress has been associated with GC-to-TA transversions which occur following association with 8-oxoG (Nakabeppu, 2014), and increased prevalence of single-strand breaks (SSBs) and double-strand breaks (DSBs), with DSBs being proxy for the following mutations: translocations, deletion and gene amplification (Trenner and Sartori, 2019). Damage to DNA and cell cycle machinery promotes genomic instability, a hallmark of cancer (Sonugür and Akbulut, 2019). It is no wonder DNA repair genes are prevalent (more than 100 genes) which maintain DNA integrity and prevent neoplastic transformation (Waters et al., 2013). Tumors additionally present with genetic instability noted as aneuploidy (Schulze and Petersen, 2011), chromosomal, intrachromosomal, microsatellite instability and even epigenetic instability (Sonugür and Akbulut, 2019), which all enhance tumor survival. *BRCA2* mutations are, for example, linked to survival pressures that mediate immune responses including CD8^+^ and macrophage infiltration, and enhance the vascularity of the stroma (Danenberg et al., 2022). That these phenomena are additionally associated with hypoxia (Sørensen and Horsman, 2020; Zarrilli et al., 2020), highlights the importance of selective pressures that drive the innate and adaptive immune system toward pro-tumor effects.

The hypoxic TME, in addition to acting directly on tumor cells, can transform stromal cells as identified by the presence of cancer-associated fibroblasts (CAFs) in breast cancer. These cells are themselves highly heterogeneous, being derived from multiple cell types. Such interaction reflects cooperative, collective behavior, whereby normal functions of fibroblasts are subverted to facilitate tumor progression (Tarabichi et al., 2013). Factors derived from CAFs and cancer-associated adipocytes (CAAs), including IL-6 and TGF-β, are associated with increased inflammation and remodeling of the extracellular matrix, EMT and limiting the recruitment of anti-tumor T lymphocytes while simultaneously shifting macrophages towards a pro-tumor phenotype (Neuzillet et al., 2015; Zarrilli et al., 2020; Tan and Naylor, 2022). These paracrine interactions facilitate disease progression by permitting the attainment of fitness advantages within the TME, and by assisting in preparing the pre-metastatic niche for colonization. The success of circulating tumor cells (CTCs) is predicated on homing to the pre-metastatic niche and protection within the harsh landscape of the bloodstream (Tan and Naylor, 2022).

While the make-up of cells within the TME has been unraveled, the interactions of these cells and their arrangement in space are neither well characterized nor their influence on response to therapy, well understood (Danenberg et al., 2022); moreover, under the influence of transformed cells, the TME itself is dynamic. The extent of immune cell infiltration and the subtypes thereof, proximity to tumor cells or stromal cells, association with invasive front, and vascularity are all parameters that have potential prognostic significance (Larsson et al., 2020). A study using imaging mass cytometry (IMC) of breast tumors from 693 patients part of the METABRIC study, uncovered the relationship between defined TME “structures,” genomic features and clinical outcomes (Danenberg et al., 2022). The findings revealed spatial intratumoral heterogeneity, or microniches, classifying ten main recurrent TME structures, including quiescent vascularized stroma and variants of immune active subpopulations, that were distinct across tumor subtypes (Danenberg et al., 2022). This heterogeneity is underwritten by phenotypic plasticity and the capacity for tumor cells to evolve in response to the selective pressures exerted by the TME. Notably, while single-cell transcriptomics of tumor cells describe the major molecular subtypes, the TME provides the most diversity comparatively, thus highlighting its role in promoting the selection of certain genes, influencing the tumor phenotype and response to therapy (Padh, 2004; Li et al., 2021; Danenberg et al., 2022).

## Genomic influences and tumor evolution in breast cancer molecular subtypes

Genomic rearrangements and driver mutations are shared by most tumor clones, illustrating that such events may occur early in the evolutionary process. Adaptations that drive intratumoral heterogeneity are considered responsible for subtype switches, and the variance observed between disseminated tumor cells and those of the primary tumor (Sato et al., 2016). Disease progression of tumor subtypes is now being further elucidated by multiple studies that have highlighted the importance of TME constituents (Danenberg et al., 2022). In line with clinical and histological findings, ER-, ER+, and HER2-overexpressing breast cancer subtypes show considerable variability in the spatial organization and components of the TME (Nagy et al., 2021; Danenberg et al., 2022). We suggest that this may represent ecological microniches that permit clonal adaptations under selective pressures, providing a background on which intratumoral heterogeneity can result. This concept is further highlighted in luminal A tumors, for example, where regions of dysfunctional T cells with expression of immune checkpoint inhibitors, e.g., PDL-1, are depleted compared to other regions within the tumor (intratumoral heterogeneity); and compared to other subtypes (intertumoral heterogeneity) where areas rich in T regulatory lymphocytes and proliferating cells are abundant, for example, in ER-tumors (Danenberg et al., 2022). Such regions were also found to have the most abundant mutations in *BRCA1* and *Casp8* (Danenberg et al., 2022). *BRCA1* mutations play an important role in facilitating genomic instability by impairing the repair of double-stranded DNA breaks through homologous recombination, resulting in a diverse genomic profile and may also facilitate robust adaptive immune response (Nagy et al., 2021), echoing its association in areas rich in regulatory T cells, the drivers of immunosuppression and tumor progression (Van Dalen et al., 2019; Li et al., 2021). Similarly, *Casp8* mutations found in regions of dysfunctional T cells inhibit Fas/FasL apoptosis driven by cytotoxic T-cells, further enhancing immunosuppression and shaping the ecology of the TME for tumor progression (Danenberg et al., 2022).

In ER+ tumors, a poor prognosis can be predicted by areas of the TME characterized by granulocyte and APC enrichment, increased levels of macrophages and T regulatory cells, and dysfunctional T cells (Danenberg et al., 2022). In contrast, vascular stroma in ER+ tumors is associated with favorable outcomes (Danenberg et al., 2022), which is of interest, given that factors associated with angiogenesis and platelet involvement tend to be associated with aggressive tumor behavior and worse outcomes (Li et al., 2014; Mezouar et al., 2016). The redefining of spaces within the TME provides an avenue for the induction of phenotypic heterogeneity in response to spatiotemporal cues. While Danenberg and others found no defined structures to be significantly associated with ER-tumors; this was attributed to lower statistical power (Danenberg et al., 2022). In ER-tumors, TILs have been shown to modulate progression, with NK cells associated with good prognosis (Tian et al., 2016), and regulatory T cells associated with tumor aggressiveness (Zarrilli et al., 2020; Danenberg et al., 2022).

Predictive modelling based on molecular subtyping and transcriptomics is further able to illustrate the risk of tumor recurrence (Belizario and Loggulo, 2019). Integrative subtyping using the METABRIC dataset is being put forward as a method by which to predict late relapse. ER-patients had a higher risk of distant recurrence and mortality within the first 5 years post-surgical intervention, whereas ER+ patients had a longer risk period (Rueda et al., 2019). Those groups at greatest risk of relapse were characterized by enrichment in genomic-copy-number alterations including *CCND1*, *MYC*, *FGFR1* and *FGF3*, and the mTOR effector *S6K1*, to name but a few (Rueda et al., 2019). Not only do such studies illustrate the limitations in current diagnostic procedures, but also in predicting recurrence with clinical markers alone; highlighting possible driver mutations and passenger mutations that can be therapeutically targeted and further stressthe need to study interactions between different components within the TME.

The diversity in tumor subtypes which arise from intrinsic molecular heterogeneity and influenced by TME composition has been reflected in significantly higher mortality rates in Black or African American women due to the prevalence of HR- or TNBC (Huo et al., 2017). It has generally been observed that while breast cancer prevalence is lower in African countries, the mortality rate is inversely higher; reflecting the prevalent tumor subtypes which are more aggressive and associated with poor prognosis (Huo et al., 2017). Among other factors such as late-stage diagnosis and low-resourced settings, in women from West Africa, the prevalence of TNBC was associated with the Duff-null allele (DARC/ACKR1) (Lal et al., 2017), conserved for its role in protection against malaria (Leong et al., 2022). This shows the intricate co-dependency of environmental and microenvironmental cues that influence the natural selection of a marker against one disease (malaria) predisposing an individual to another disease (cancer) (Leong et al., 2022). Expression of the *CRYBB2* gene and other lipid-metabolism genes have also been found to differ between people with African and European ancestry (Huo et al., 2017). Such data reveals the underlying molecular rationale between disproportionate mortality rates between population groups; moreover, it indicates that personalized risk assessment may be key to defining optimal treatments to reduce breast cancer deaths in black women.

## Some precision technologies for the diagnosis and treatment of breast cancer

Several emerging technologies have been utilized in the diagnosis and monitoring of breast cancer patients. We highlight several of these diverse technologies and their relevancy, describing recent efforts to assess them in research and the clinic. We suggest to the reader that the utility and efficacy of these technologies require contextual consideration of tumor evolutionary dynamics in relation to the tumor ecosystem—the TME.

## Next-generation sequencing

Next-generation sequencing is commonly used to identify genes and mutation hotspots (Hamdan et al., 2019). Pan-cancer analyses using NGS are critical in unravelling cancer genes and candidate cancer genes, with the majority of these having arisen in pre-metazoan species (Repana et al., 2019). Recently, the emergence of single-cell NGS has enabled the delineation of the intricate characteristics of the TME at a molecular level (Ren et al., 2021; Tan et al., 2022). One study identified 9 “ecotypes” with unique characteristics resulting in different clinical outcomes (Wu et al., 2021). This highlights the interdependence of tumor cells on cells of the TME, with tumor clones capable of creating ecological niches from which they can enhance their fitness and undergo dispersal (invasion), dependent on their evolvability. Whole genome and exome sequencing has been useful in resolving various types of somatic and germline mutations on a larger scale (Hamdan et al., 2019). Various types of mutations are detected through whole genome sequencing including single nucleotide variants (SNV), indels (insertions or deletions) and structural variants (SV) (Hamdan et al., 2019). Whole genome sequencing has revealed heterogeneity among the mutation landscape that defines each tumor type. Some mutations within a tumor occur in a certain clone or cells, whereas different types of tumors show distinct mutational profiles which uniquely identify them, presenting a further challenge in the clinical management of the disease. Using mutational signatures and mathematical modeling, coupled with machine learning techniques, algorithms are being developed to more accurately predict causative factors, prognosis and treatment strategies (Davies et al., 2018; Alexandrov et al., 2020).

Next-generation sequencing has led to the identification of prominent genes such as *BRCA1* and *BRCA2* involved in breast cancer initiation and progression. *BRCA1*, involved in homologous recombination, is one of the most commonly mutated genes in hereditary breast cancer and TNBC (75%) (Gonzalez-Angulo et al., 2010). Constitutional BRCA1 mutations occur in 10% of breast cancer patients and 20% in younger women (Peto et al., 1999). One percent of breast cancer present with sporadic mutations in *BRCA1* with the promoter region in the gene being hyper-methylated in 11%–14% of cases thus inactivating the gene (Rigakos and Razis, 2012; Leidy et al., 2014). Sophisticated algorithms like the HRDetect tool can accurately detect *BRCA1/BRCA2* deficiencies with therapeutic potential (Davies et al., 2018). A study conducted on a small cohort of TNBCs illustrated the utility of single-cell DNA sequencing showing that genomic-associated resistance to neoadjuvant chemotherapy was evident in pre-existing clones; however, the addition of single-cell RNA sequencing highlighted transcriptional reprogramming as an adaptive response to therapy (Kim et al., 2018). This highlights how critical it is to use multiple assays to understand tumor responses in light of evolutionary mechanisms that impact treatment efficacy. Another study investigated the efficacy of next-generation sequencing in detecting mutations in circulating DNA via liquid biopsies and the corresponding tumor mass in 75 women diagnosed with early-stage breast cancer (Jiménez-Rodríguez et al., 2023). The findings showed that mutations commonly occurred in *TP53*, *PIK3CA* and *GATA* genes, in liquid biopsies and the corresponding biopsies, corroborating other studies and data in the TCGA database (Jiménez-Rodríguez et al., 2023). In some cases, there was a lack of correlation in mutations between plasma samples and corresponding tumors, thus highlighting intratumoral heterogeneity (Jiménez-Rodríguez et al., 2023). These findings showed the efficacy of liquid biopsies with the application of NGS techniques in detecting the heterogenous nature of the tumor landscape in early-stage disease and may contribute to the establishment of better disease management strategies which are targeted at heterogenous cell populations. However, this also highlights that temporal adaptations in tumor cells and in the TME need to be assessed to determine their impact on metastatic disease, recurrence and relapse.

### Assessing NGS technologies using gene panels/tests

Using NGS technologies, the prevalence of genes associated with increased risk of breast cancer is well established. With an increase in the use of sequencing technologies, several novel variants are being discovered; however, their significance is yet to be established (Easton et al., 2015). These variants are frequently being discovered in high-risk genes, especially with a gradual increase in testing across different ethnicities and populations. Recently, 77,900 women with breast cancer were tested using a panel for germline pathogenic variants. It was found that the occurrence of these variants differed according to ethnicity. For instance, *BRCA1* mutations occurred more frequently in Hispanics and Ashkenazi Jews compared to non-Hispanic white patients (Yadav et al., 2021). Studies like this highlight the need for a personalized approach to the diagnosis of patients regarding the development and use of multigene panels across different ethnicities and population groups, and the interpretation of the results thereof. For example, in Africa, there is generally little genomic data from cancer patients. A study by Rotimi *et al* found that only 0.329% of cancer genomics studies have been conducted in African patients. Regarding breast cancer, most of the work looked at mutations in *BRCA1* and *BRCA2* with several studies reporting novel variants in these genes (Rotimi et al., 2021). However, *BRCA1/2* mutations constitute but a small part of breast cancers, approximately 25% of patients with TNBC, which itself is said to constitute about 15% of all breast cancers presented (Barchiesi et al., 2021). Population differences have also been identified in which African Americans were found to have more *TP53* mutations and fewer *PIK3CA* mutations than Americans of European ancestry. Significant clinical outcomes were found between population groups, even after controlling for intrinsic subtype frequency differences, highlighting that further risk assessments need to be conducted to improve outcomes for black women (Huo et al., 2017). Therefore, this restates the need for more population-based studies to be conducted to identify and establish the potential significance of these variants to better inform genetic counselling and management of patients.

### Clinically relevant gene panels/tests

In recent years, there has been widespread interest in the clinical utility of gene panels/tests for breast cancer detection and risk assessment. Several studies have intricately analyzed the utility of these panels in clinical settings (Easton et al., 2015; Lerner-Ellis et al., 2015; Catana et al., 2019; Piccinin et al., 2019). Available panels have been extensively reviewed and it has been found that the occurrence of pathogenic variants (PVs) was highest in *BRCA1* followed by *BRCA2*, *CHEK2*, *PALB2* and *ATM* (Lerner-Ellis et al., 2015). A multicenter study used a 34 multigene panel and screened more than 60,000 women with breast cancer. The authors identified protein-truncating variants in *BRCA1*, *BRCA2*, *CHEK2*, *PALB2* and *ATM* that were significantly associated with breast cancer risk (Breast Cancer Association Consortium et al., 2021). Another study demonstrated the utility of multigene panels by utilizing screening samples obtained from 35,409 women with breast cancer against 25 genes. They observed that using this panel increased the identification of patients with PVs. Importantly, they found that 51.5% of the PVs were found in genes, not including well-known ones (*BRCA1*, *BRCA2*, *CHEK2*, *ATM*, *PALB2*), that are associated with increased risk (Buys et al., 2017).

Some of these panels have been used in theranostics whereby they have been applied for diagnosis and the identification of therapeutics to be administered. Some of them have been developed into commercial tests that can be used in clinical settings. Their clinical utility can include detection, prediction, treatment decision-making, monitoring relapse and response to treatment (Lal et al., 2017). For instance, some of these tests can quantify specific genes which would predict metastasis relapse and thus be useful in determining the type of adjuvant chemotherapy in ER+ and HER2-patients without lymph node involvement (Sotiriou et al., 2006; Rakha and Green, 2017). Examples of the tests include MapQuant DX ™ (Genomic Grade Index, GGI) (Sotiriou et al., 2006), Pro-Signa® (Nielsen et al., 2014; Wallden et al., 2015), Mammaprint® (Wittner et al., 2008), Oncotype DX® (Paik et al., 2004), BluePrint® (Mittempergher et al., 2020) Endopredict® (Filipits et al., 2011). Several ongoing clinical trials are assessing the efficacy of these gene panels in the diagnosis and prognosis of breast cancer (Table 1).

**TABLE 1:** Some ongoing clinical trials utilizing various gene panels/tests.

Gene panel/test	Number of genes	Utility	Ongoing (recruiting) clinical trials
PAM-50 (Pro-Signa) Wallden et al. (2015)	50	Prediction and risk assessment	19- including NCT04344496, NCT03749421, NCT04759248, NCT03769415, NCT02889874, NCT01560663, NCT04578106, NCT03904173, NCT02448420, NCT04610528, NCT04759248
MammaPrint® Wittner et al. (2008)	70	Prediction and prognosis	4- NCT05474391, NCT03053193, NCT03900637, NCT04129216
EndoPredict® Filipits et al. (2011)	12	Prognosis	4- NCT04246606, NCT03503799, NCT03969121, NCT01805271
BluePrint® Mittempergher et al. (2020)	80	Diagnosis	4- NCT03053193, NCT04129216, NCT05252416, NCT03900637
Oncotype DX® Paik et al. (2004)	21	Prediction	11- NCT04852887, NCT03495011, NCT02476786, NCT01560663, NCT03212170, NCT03961880, NCT03703492, NCT02095184, NCT04797299, NCT02993159, NCT03220893
Invitae https://www.invitae.com/en/providers/test-catalog/test-01102	8 different kits with up to 84 genes	Prediction, risk assessment	2- NCT04354675, NCT04985266

### Assessing NGS technologies using artificial intelligence and machine learning

The growing incidence of breast cancer and the accumulation of data produced by technologies such as NGS and the potential variability between specialists such as pathologists have necessitated the use of computational models such as artificial intelligence (AI) and machine learning to enhance breast cancer diagnosis (Aruleba et al., 2020; Bhinder et al., 2021). As the understanding of the TME grows, more emphasis is being placed on immune-associated genes that could act as prognostic indicators, as well as other markers associated with the hallmarks of cancer (e.g., proliferation, angiogenesis) which may hold genomic information that may impact therapeutic management (Kudelova et al., 2022; Lin et al., 2022; Zhao et al., 2023). For example, artificial intelligence is being used to generate immune signatures of tumors to differentiate between immune-related breast cancer subtypes (Thomas et al., 2021). Algorithms such as CIBERSORT and ImmuneScore can further be used to determine relative immune cell abundance and define populations that may drive progression or have predictive value (Lin et al., 2022). Multitiered spatial analysis of tumor samples is additionally showing how genomics could help stratify patients and impact care (Danenberg et al., 2022). These ‘big’ datasets reveal intratumoral heterogeneity, while simultaneously illustrating that tumors derived from different organs nevertheless may share underlying features (Bechtel, 2019). Pathway analysis and network clustering assist in drawing together features that may be linked to the defined hallmarks of cancer, to assist in explaining tumor progression, but may be limited by, for example, datasets derived from primary lesions that cannot fully recapitulate the adaptations that occur in metastatic clones or locoregional relapse (Yates et al., 2017; Bechtel, 2019).

Deep convolution neural networks have also been adapted for use in the identification and classification of tumor-associated stroma using biopsies obtained from breast cancer patients. For example, an algorithm was trained to discriminate benign tissues from cancerous tissue, adequately classifying both groups by analyzing their stromal content (Ehteshami Bejnordi et al., 2018). Wu and Hicks, using data obtained from The Cancer Genome Atlas (TCGA), evaluated 4 different machine learning algorithms and observed that utilizing a support vector machine algorithm resulted in a more accurate classification of triple-negative tumors from other breast tumors with few errors (Wu and Hicks, 2021). In another study using data repositories such as TCGA, the authors accurately differentiated between hormone and HER2 receptor phenotypes using a median-supplement method with precision and low false positives (Adabor and Acquaah-Mensah, 2019).

A systematic review over a 5-year period (2015–2019) found 2928 publications based on machine learning for breast cancer detection, concluding that this area of research is fast-growing with potential clinical utility (Salod and Singh, 2020). CAMELYON16 demonstrated that in a time-constrained environment, deep learning algorithms could outperform pathologists in the accuracy of detection of metastasis in breast cancer lymph nodes (Ehteshami Bejnordi et al., 2017). This type of technology could be especially beneficial in regions with an increased burden of the disease and few expert pathologists such as in some low-to-middle-income countries.

## Proteomic technologies

Proteomic approaches, especially array and sensor-based technologies, have been employed in several breast cancer studies to identify biomarkers, and elucidate tumor heterogeneity. The identification of the specific protein to be assayed and integrated into these targeted technologies, are usually a result of studies that have applied a global approach to proteomic profiling to diverse biosamples including tissue, blood, urine and tear-fluid. One of the most widely used technologies for global proteomic profiling is mass-spectrometry (MS)- based (Neagu et al., 2022). A recent study used mass-spectrometry to profile 300 FFPE breast cancer tissues and efficiently characterized heterogenous subtypes including linking them to clinical outcomes (Asleh et al., 2022). More targeted proteomic technologies such as IHC, ELISAs, and more recently antibody arrays including reverse-phase protein array (RPPA) and immunosensors have been utilized in breast cancer diagnosis (Neagu et al., 2022). The clinical utility of RPPA have been demonstrated using FFPE breast cancer tissues, showing it to be a robust and highly reproducible technique for the identification of 10 well-known biomarkers of breast cancer with results obtained comparable to IHC (Assadi et al., 2013; Negm et al., 2016). Due to the growing interest in the use of RPPA for biomarker identification in breast cancer, extensive workflows have been developed a point-of-care breast cancer detection technology (Sonntag et al., 2014; Coarfa et al., 2021). This means that breast cancer diagnosis can be conducted fast and easily, especially in environments with limited access to laboratory or research centers. Most of these immunosensors/arrays were developed to detect multiple tumor biomarkers which could increase their specificity for the detection of breast cancer (Hasanzadeh et al., 2017). A novel label-free electrochemical immunosensor was developed using the tumor markers CA125, CA15.3, and CEA, and was demonstrated to exhibit optimal sensitivity, accuracy and reproducibility in detecting breast cancer (Cotchim et al., 2020). Another fast, easy and convenient detection method including the same tumor markers was developed by Ge and others whereby they used a disposable electrochemical array which could eliminate cross-talk and does not require deoxygenation (Ge et al., 2012). In another study, a high-density silicon array was utilized to capture and quantify EGFR2 (limit of detection = 25 ng/mL); moreover, this array was designed to be wearable, making it an accessible device and reiterating its potential to be used as a point-of-care detection tool (Dervisevic et al., 2021).

Utilizing the principle of multiple sensoring, breast cancer biomarkers (CA15.3 and HER2), were detected at levels lower than the accepted cut-off in clinical settings. In human serum, the biosensor showed higher sensitivity, and selectivity with reproducible results (Kuntamung et al., 2021). The simultaneous detection of HER2 and CA15.3 in breast cancer patients has also been made possible by the development of a voltammetric immunosensor providing another non-invasive and sensitive approach to detection (Marques et al., 2018). The detection of tumor-associated molecules or biomarkers in serum or other bodily fluids, would provide a rapid, non-invasive method to track the evolvability of tumor cells, regarding disease stage and response to treatment. During the metastatic process, vascular components and the high shear forces within the vasculature, for example, induce phenotypic alterations in tumor cells as an adaptive measure to a vastly different and dangerous microenvironment (McDowell and Quail, 2019; Baghban et al., 2020; Tomita, Kato, Hiratsuka, 2021). The capacity to monitor a range of biomarkers, in concert with clinical data, in real-time may permit more effective patient management.

## Microfluidics platforms

Microfluidic technologies provide an avenue to rapidly detect breast cancer at its early stages thereby potentially improving patient outcomes (Panesar and Neethirajan, 2016). In one of the first studies to do so, Kim and others used a microfluidic platform to detect breast cancer employing the detection of several biomarkers, accurately and simultaneously in small tissue sections, to provide a histopathological diagnosis (Kim et al., 2010). A low-cost microfluidic chip using an electrical double-layer capillary capacitor showed potential clinical value in diagnosing breast cancer by detecting CA153 at a low limit of detection of 92 μU/mL, suggesting its sensitive nature (de Oliveira et al., 2018).

Circulating exosomes and extracellular vesicles (EVs) derived from tumors in several cancers, including breast cancer, can be quantified for early detection and evaluation of the risk of metastasis. The detection, quantification and molecular classification of breast cancer was demonstrated using a microfluid chip (Fang et al., 2017). Specifically, EPCAM-positive and HER-positive exosomes were quantified showing similar expression levels in tissue samples. A similar study utilizing circulating exosomes also demonstrated the use of microchip technology in detecting EPCAM showing a 90% and >95%, sensitivity and specificity, respectively (Chen et al., 2019). Another study used a microfluidic chip to detect EVs expressing EPCAM and CD49F, both epithelial and mesenchymal markers, respectively. Their analysis demonstrated that patients with a high EMT index of ≥5 detected by the microchip, underwent recurrence within 5 years (Gwak et al., 2021). EVs-encapsulating microRNAs can also be detected with a microfluidic chip. MicroRNAs are known to regulate the expression of crucial genes and thus can be effectively utilized as disease biomarkers. Using DNA-FET biosensors linked to a microfluidic system, microRNA-195 and microRNA-126 were both detected and quantified in breast cancer samples (Huang et al., 2021). Typically, dysregulation of miRNAs is associated with neoplastic transformation and may provide a platform for increased cellular entropy, favoring evolvability and the capacity for tumor cells to escape physiological controls (Tarabichi et al., 2013; Pienta et al., 2020). Microfluidics assessment of shed microvesicles would provide a non-invasive and real-time assessment of tumor cell adaptation and resistance to therapy.

## Therapeutic targets and associated therapies in breast cancer

The ability to identify the molecular underpinnings of a tumor allows for precise targeting to increase the chances of effectiveness and mitigate some adverse effects and collateral tissue damage that may influence recurrence or resistance to treatment. We propose that for more effective therapy, consideration must be given to the concept of speciation in cancer, whereby the generation of distinct clones predicated on the initial capacity of transformed cells to adapt to microenvironmental stressors or evolvability, leads to a heterogenous mass with clones that may either have an intrinsic resistance or develop an adaptive resistance, to therapies (Pienta et al., 2020; Bukkuri et al., 2023). Moreover, in the context of the TME, while therapies may target specific tumor-related antigens, few therapeutic regimens exist that would prevent evolutionary rescue of the ‘cells left behind’ that ultimately drive recurrence and resistance. In this section we note advances in selected targeted therapies and consider the challenges in their approach.

### HER2

**FIGURE 2:**
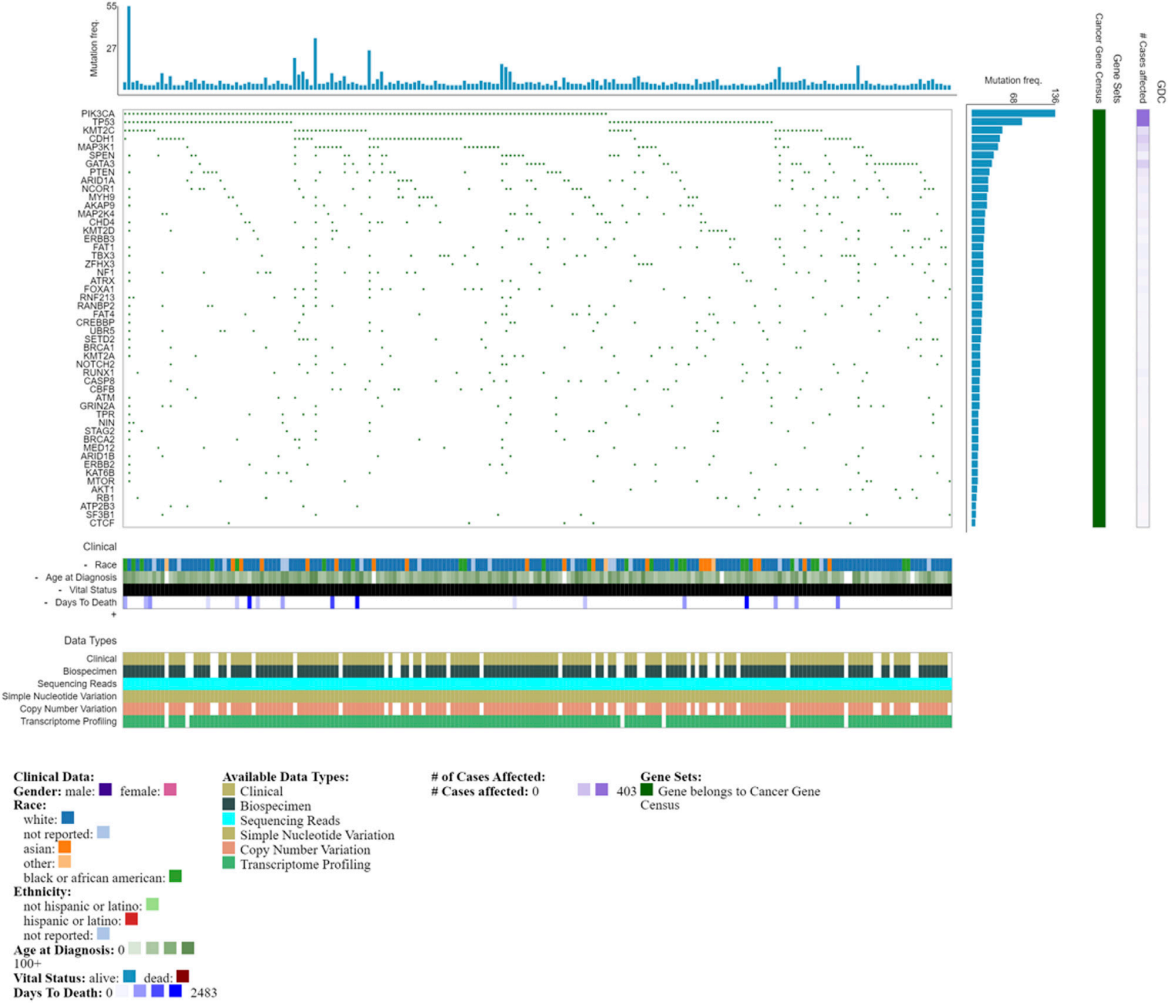
Oncogrid of the top 200 most mutated cases and top 50 mutated genes by simple somatic mutations. Data obtained from NCI Genomic Data Commons (GDC). Data accessed on the 31st October 2022.

Several HER2 monoclonal antibodies have been developed for use in HER2-enriched breast cancer including trastuzumab and pertuzumab (Yu et al., 2017). HER2 plays a critical role in driving tumor formation and progression, by forming homodimers and heterodimers with members of its receptor tyrosine kinase family including HER1/EGFR and HER3. Additionally, HER2 initiates downstream signaling in the PI3K/Akt/mTOR and MAPK pathways (Yu et al., 2017), which are frequently dysregulated in breast tumors (Figure 2). Trastuzumab functions through blocking homodimerization of HER2, whereas pertuzumab blocks heterodimerization (Yu et al., 2017). This leads to good clinical response rates, although some patients show *de novo* and acquired resistance (Yu et al., 2017). Accurate and repeatable characterization of HER2 tumors including HER2-low tumors is becoming increasingly important to determine the most beneficial treatment (Baez-Navarro et al., 2022). The loss of HER2 is a mechanism of acquired resistance for treatments targeting this receptor. The efficacy of Trastuzumab is affected by the immune cell milieu, with effective therapy requiring a mounted Th1 response (Costa and Czerniecki, 2020; Li et al., 2021). Such therapy may lead to antibody-dependent cellular cytotoxicity (ADCC) by natural killer cells and cytotoxic T lymphocytes, thus advances in drug development are aimed at enhancing the anti-tumor immune response (Costa and Czerniecki, 2020). Combined use of pertuzumab, trastuzumab and paclitaxel is a standard treatment regimen, but there is considerable risk for the development of trastuzumab resistance (Yu et al., 2017). This has led to the development of HER2-bispecific antibodies that have yet to be fully evaluated clinically.

Antibody-drug conjugates (ADC) comprise a HER2-antibody coupled with a cytotoxic agent, that following uptake and release by targeted cells, has a limited capacity to diffuse to antigen-negative cells and induce cytotoxicity (Costa and Czerniecki, 2020). T-DM1 was the first FDA approved ADC for use in HER2-enriched tumours, and consists of trastuzumab conjugated to a naturally occurring microtubule polymerization inhibitor, emtansine (Peddi and Hurvitz, 2014). Following the EMILIA study, T-DM1 was approved for use in HER2-enriched breast cancer patients previously treated with Trastuzumab and a taxane (Hunter et al., 2020). T-Dxd was the second FDA approved ADC and consists of a humanised HER2-targeted antibody with similar structure to trastuzumab, conjugated with a topoisomerase I inhibitor, Dxd. T-DXd also acts on neighboring cells which may not express HER2 through the bystander effects, accounting for its efficacy in HER2-low tumors (Popović et al., 2023). These treatments are associated with adverse effects including high-grade thrombocytopenia and interstitial lung disease (Najjar et al., 2022). There are several ADC therapies which are in clinical trials as novel agents or an improvement of existing therapies that have shown efficacy mostly in metastatic HER2-enriched breast tumors including SYD985, ZRC-3256, TAA013 all in phase III trials; MRG002 in phase II trials; A166 and SHR-A1201 in phase I/II trials; and ALT-P7, ZW49, Bi-CON-02 and B003 in phase I trials (Najjar et al., 2022; Yu et al., 2022). Certain ADC therapies for HER2-enriched breast tumors have shown promising efficacy against HER2-low tumors including trastuzumab-duocarmazin (SYD 985), disitamab-vedotin (RC48) (Shi et al., 2022) and durvalumab and T-Dxd (Popović et al., 2023). Other ADCs are being developed that are considering reduced toxicity profiles as well as engaging anti-tumor immune cell response via the FcγR (Costa and Czerniecki, 2020). Several small-molecule tyrosine-kinase inhibitors (lapatinib, neratinib and pyrotinib) have been developed aimed at interrupting the HER signaling pathway and preventing downstream pro-tumor effects (Dong et al., 2021).

Studies have additionally highlighted conversion between HER2-low subtype and HER2-enriched subtype, with the latter identified in brain metastasis (Turner et al., 2021); and patients with luminal A subtypes that differentiate into HER2-enriched metastatic cancer but remain unresponsive to therapy (Garcia-Recio et al., 2020). Implicated in such conversions would be microenvironmental cues that shape the responses of selected clones to and within colonized tissue, notably with spatiotemporal evolution at the core of producing heterogenous variants.

### PARP inhibitors

TNBC display considerable heterogeneity with up to 25% of patients diagnosed carrying germline *BRCA1*/*BRCA2* mutations (Barchiesi et al., 2021). Patients with *BRCA2* variants are also likely to develop ER+ tumors (Tutt et al., 2021). Tumor cells that carry *BRCA1/2* variants are dysfunctional in the homologous recombination repair pathway. Poly (ADP-ribose) polymerases (PARP), are a family of enzymes that play a fundamental role in base excision repair, by detecting DNA damage and catalyzing the repair itself (Barchiesi et al., 2021). Furthermore, PARP is involved in mediating stress response, chromatin remodeling and apoptosis ((Rose et al., 2020). Tumor cells use PARPs to ensure increased entropy, accumulating genomic alterations that drive genomic instability that can then be passed down to progeny. PARP inhibitors (PARPi) exert their anti-tumor function by competitively displacing NAD + to bind to the catalytic site of PARP1 and PARP2, thus preventing DNA repair and thereby driving tumor cell death (Rose et al., 2020). PARPi may also induce tumor cell death by inhibiting PARylation mechanisms, effectively trapping DNA on PARP with the resulting accumulation of damage responsible for the cytotoxic effect (Barchiesi et al., 2021). The capacity of PARPi to induce cell death via these modalities differs according to the drug employed. Currently, PARPi, olaparib (OlympiAD trial) and talazoparib (EMBRACA trial) have been approved for clinical use in HER2-, metastatic breast cancer, where patients carry the *BRCA1/2* variants (Tung and Garber, 2022). Additionally, such therapies allow healthy cells to be spared, a distinct clinical advantage; however that 50% of patients progressed during treatment. This highlights the need to investigate the mechanism of either treatment failure or the development of resistance (Barchiesi et al., 2021). Implicated in resistance, is the persistence of disparate tumor clones with intrinsic resistance to a therapy or the development of clones with acquired resistance that arises due to adaptation to therapy (Nagelkerke et al., 2014).

Concerns regarding acquired resistance have been noted using PARPi in breast cancer with *BRCA1/2* mutations where the overexpression of drug-efflux transporters and the occurrence of reverse mutations may lead to the reactivation of BRCA1/2 function and the restoration of the homologous recombination repair pathway (Barchiesi et al., 2021; Tung and Garber, 2022). Nevertheless, the efficacy of PARPi was also shown in patients without genomic *BRCA1/2* mutations and high genome-wide loss of heterozygosity (LOH) score, albeit in a smaller proportion of patients (Chopra et al., 2020; Patsouris et al., 2021). Notably, Davies and others (Davies et al., 2018) developed the HRDetect predictive tool which detects *BRCA1* and *BRCA2* deficiencies at a level of 98% sensitivity, and using mutational signatures to identify a much greater number of patients (from 1% to 5% previously, up to 22%) who could benefit from PARPi therapy.

PARPi has been tested as part of a combined therapy regimen to minimize drug resistance. Alkylating agents combined with PARPi improve patient survival but when combined with topoisomerase I inhibitors no significant benefit was detected (Rose et al., 2020). PI3K inhibitors heightened the sensitivity of TNBC to PARPi, similarly, increased efficiency of radiotherapy was observed when combined with PARPi and anti-tumor immune response was evident. This highlights the need for further investigations into these therapy combinations. In addition, possible adverse drug metabolism has to be investigated further as PARPi (specifically Olaparib) are major substrate for CYP34A enzymes in the liver (Menezes et al., 2022). The efficacy of Olaparib was shown to be reduced when used concomitantly with other drug agents which are substrates for CYP3A4 enzymes (Menezes et al., 2022).

### Immune checkpoint inhibitors

TILs have been suggested to have predictive and prognostic values in breast cancer treatment and management (El Bairi et al., 2021). A study involving 2148 patients with early-stage TNBC from 9 different studies proposed the prognostic utility of TILs and showed that elevated levels of stromal TILs were linked to patient survival (Loi et al., 2019). TNBC is regarded as a more immunogenic breast cancer, highlighted by increased levels of genomic instability, raised expression of PD-L1 and greater responsiveness to immune checkpoint inhibitors (Barchiesi et al., 2021). Breast cancer has a lower mutational load than other tumors, and thus produces lower quantities of neoantigens than, for example, melanoma, and is thus less likely to elicit a T cell immune response. However, in TNBC not only is the T cell infiltration higher than in HER2-overexpressed and ER+ tumors, but so too the mutational load (Hammerl et al., 2018). Mutational load in ER+ BC cancers, is also shown to increase in higher grade tumors, but whether this is associated with increased immunogenicity remain to be unraveled (Hammerl et al., 2018). Nevertheless, even with ER-positive breast cancer patients TILs score have a strong prognostic value (He et al., 2021), with subsets thereof being more or less likely to be associated with good prognosis. For example, high frequencies of CD8 effector T cells and Th1 helper T cells are correlated with good clinical outcomes, particularly in ER-tumors. However, while T regulatory cells are associated with good prognosis in ER-tumors, in ER+ tumors they are linked to poor clinical outcomes (Hammerl et al., 2018). It is increasingly evident that immune cell subsets, in addition to TILs require tracking in disease and standard therapy. For example, after neoadjuvant therapy, the levels of residual disease TILs were associated with overall survival and recurrence-free survival (Luen et al., 2019). Due to the observed role of TILs in breast cancer progression, albeit not completely well-defined, and an understanding of the immunoediting hypothesis, several immune checkpoint inhibitors against PD-1, PD-L1 and CTLA-4 have been tested in the treatment of the disease (Rizzo and Ricci, 2022). These co-inhibitory signals prevent the engagement of the immunological synapse and subsequent destruction of target cells (Voutsadakis, 2016). PD-1 is typically expressed on NK cells, T and B lymphocytes while its corresponding ligand is expressed on APCs. In cancer, however, PDL-1 and PDL-2 are overexpressed on tumor cells and postulated to induce anergy or apoptosis of these lymphocytes and promote T regulatory immunosuppression (Salemme et al., 2021). This manipulation of the TME shows cooperative behavior between populations, leading to adaptive responses that enhance fitness, in the case of the tumor cells at least. Greater PD-L1 expression has been observed in HER2-enriched tumors and TNBCs, providing potential targets for these aggressive tumors (Salemme et al., 2021; Thomas et al., 2021). CTL4, a co-stimulatory receptor on T lymphocytes, is also upregulated in breast cancer cells. While its function is not completely known in cancer contexts, it has been shown to enhance PD-L1 expression, and thus create an immunosuppressive TME (Salemme et al., 2021).

A meta-analysis included 3,612 breast cancer patients encompassing 6 clinical trials, evaluated the efficacy of Atezolizumab (PD-L1 inhibitor) and Pembrolizumab (PD-1 inhibitor) in breast cancer treatment. The study concluded that combination with chemotherapy was best in improving patient outcomes, especially in PD-L1-positive patients (Latif et al., 2022). Nivolumab, an approved PD-1 inhibitor, has shown efficacy in more advanced, unresectable cancers (Gaynor et al., 2022). A range of PD-L1 inhibitors including Atezolizumab have been engineered to prevent ADCC while simultaneously sterically blocking the interaction with its cognate receptor; and Avelumab which conversely elicits ADCC (Gaynor et al., 2022). While Atezolizumab is approved to treat TNBC, emerging therapies including KNO35 in HER2-enriched patients are underway (Gaynor et al., 2022), which aim to mitigate adverse immune responses that may result and drive tumor progression. While there is some indication for the use of PDL-1 inhibitors in ER+ breast cancers, low response rates have thus far been indicated in trials such as JAVELIN (Gaynor et al., 2022). Even in PD-L1+ TNBC the response rate (8%–20%) to PD1/PD-L1 treatment remains to be improved (Thomas et al., 2021). Combining PARPi with immune checkpoint inhibitors has been suggested to enhance anti-tumor efficacy. Ongoing trials include the TOPACIO trial for niraparib combined with pembrolizumab and the MEDIOLA trial for Olaparib combined with durvalumab (Barchiesi et al., 2021). Similarly, inhibiting HER2 signaling using trastuzumab in combination with pembrolizumab in patients regardless of PD-L1 presentation is also ongoing (Swoboda et al., 2018). The anti-CTLA-4 ipilimumab, approved to treat metastatic melanoma, is under consideration for use in combined treatments for breast cancer, to prevent T cell anergy (Voutsadakis, 2016). While studies have shown that CTLA-4 inhibition improves immune responsiveness and anti-tumor efficacy, the translational to clinical benefit needs assessment (Gaynor et al., 2022).

Novel immune checkpoint inhibitors are being assessed at pre-clinical and early-stage clinical trials. These include using drugs LAG525 and INCACN02385 to target lymphocyte activation gene-3 (LAG3) and MGD009 to target B7-H3, to prevent T cell exhaustion (Thomas et al., 2021; Gaynor et al., 2022). Other drugs being developed are aimed at enhancing immune checkpoint stimulators, for example, TRX-518 which targets GITR to stimulate T lymphocyte and NK cell activation (Gaynor et al., 2022). Challenges to such therapies include inefficient activation of anti-tumor responses for clinical benefit, and the development of resistance as tumor cells undergo adaptive changes in response to selective pressures in the TME.

### Akt/MTOR inhibitors

The PI3K/Akt/mTOR pathway is dysfunctional in many tumors, promoting the biological processes of proliferation, invasion and evasion of cell death (Miricescu et al., 2021), but drug resistance in breast cancer (Dong et al., 2021). Genetic alterations in *PIK3CA, PIK3R1, PTEN, AKT, TSC1/2, STK11* and *MTOR* can activate this pathway (Janku et al., 2018). Many inhibitory drugs are currently under development, or in clinical trials, but few have been indicated for clinical use, with barriers to translation including toxicity (Janku et al., 2018). PI3K and mTOR share structural domains, belonging to the PI3K-related kinase superfamily (PIKK). As such, dual inhibitors like BEZ235 (Dactolisib), target both active sites inhibiting events upstream and downstream of Akt (Dienstmann et al., 2014). Initially driven towards treatment of ER+ breast tumors, where up to 40% of ER+ HER-present with mutations in *PIK3CA* (Vasan et al., 2014), these therapies may also be promising particularly for TNBC for which there are fewer targeted therapies; however, given inconsistent results the clinical development of this drug has been discontinued (Janku et al., 2018). Pan-PI3K inhibitors, for example, buparlsisib (BKM120) and pictilisib (GDC-0941), can target the four different isoforms of class I PI3K (p110α, p110β, p110δ, and p110γ), are suggested to have greater efficacy in TNBC tumors which may have multiple PI3K alterations (Dienstmann et al., 2014). However, reviewed clinical trials showed significant psychiatric adverse effects with buparlsisib, and no significant endpoints were met possibly linked with investigators modulating dose regimens to minimize toxicity (Janku et al., 2018). Isoform-specific PI3K inhibitors, such as BYL719 that target the p110α, provide an option to customize therapy rather than risking cumulative toxicity, especially when considering synergistic use with other cancer treatments, for example, fulvestrant for ER+/PR+, HER2-metastatic breast cancer (Dienstmann et al., 2014; Dong et al., 2021). ER+/PR+, HER2-breast cancer treated with allosteric mTOR inhibitor, everolimus, which together with Anastrozole, an aromatase inhibitor, has shown improved overall response rates (Baselga et al., 2014). Since Akt can activate mTOR signaling which drives tumor progression, several allosteric and ATP-competitive Akt inhibitors are under development, with only MK-2206 further investigated in breast cancer (Martorana et al., 2021). Similarly, issues of toxicity, as seen in trials involving the majority of PI3K and mTOR drugs have been raised, with dose modifications due to toxicity also linked to poor efficacy (Janku et al., 2018; Martorana et al., 2021). There is also the challenge of drug resistance. PI3K and Akt can phosphorylate ERα at Ser167, activating the ER in the absence of estradiol, as such tumor cells can circumnavigate hormone therapy, adapting and undergoing estrogen-dependent growth (Dong et al., 2021). Activation of the PI3K/Akt/mTOR pathway and the loss of PTEN, implicated in DNA repair, activity is also linked with resistance to anti-HER2 therapy, driven by mutations in the p110α subunit (Dong et al., 2021; Miricescu et al., 2021). As such, PI3K/Akt inhibitors in combination with tamoxifen, or aromatase inhibitors are a developing multiplex strategy for ER+ or HER2+ breast cancer (Dong et al., 2021).

Given the importance of the PI3K/Akt/mTOR pathway in breast tumor progression, further research is required to understand the impact of such drugs on the TME, which may reduce cytotoxic effects and enhance the anti-tumor function of other standard treatment regimens. Consideration must be given to the activation of such pathways in immune cell populations of the TME (Goswami et al., 2017), this can in turn enhance cytokine-mediated crosstalk with tumor cells, thereby providing an additional, novel therapeutic target.

### Drug sensitivity screening

Drug sensitivity screening may provide an efficient way to assess the effectiveness of response to chemotherapy (Nweke and Thimiri Govinda Raj, 2021). These platforms include a panel of chemotherapeutic drugs and their efficacy against cancer cells can be assessed as a monotherapy or in combination. In one study, 37 breast cancer patients were screened using the combination therapy of 5-fluorouracil, epirubicin and cyclophosphamide (FEC), predicting the patient’s response to the drugs (Villman et al., 2005). Specifically, patients with locally advanced breast cancer with reduced drug resistance had better prognoses whereby their tumor progression was more delayed. By assessing the chemotherapeutic efficacy of a panel of 14 anticancer drugs on 100 tumor samples including breast cancer, the response of drugs *in vitro* was equivalent to the expected clinical performance (Haglund et al., 2012). The potential of drug screening tools in improving patient treatment and management has also necessitated the development of novel technologies. For example, the development of a 96well plate with built-in-micro gap demonstrated an optimum IC50 value when breast cancer cell lines, MCF7 and MDA-MB-231, and primary breast tumors, were screened against Cisplatin and Docetaxel (Ma et al., 2015). Another novel technology evaluated the use of CTCs in effective therapeutic response. This approach, which involved the integration of microfluidics and tapered microwells were integrated with microfluidics, could be used to determine patient prognosis via the evaluation of the formation of CTC clusters (Khoo et al., 2016).

## Discussion

**FIGURE 3:**
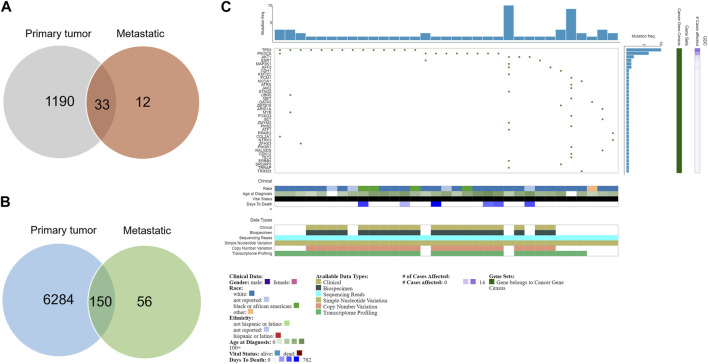
Mutations in primary and metastatic tumors. **(A)** Venn diagram of the number of cases in primary and metastatic tumors. Only 45 metastatic cases are recorded **(B)** Venn diagram of the number of mutations in primary and metastatic tumors. Fifty-six mutations are unique to metastasis patients. **(C)** Oncogrid of the top 34 mutated cases and top 38 mutated genes by simple somatic mutations that are unique to metastatic patients. Data was obtained from NCI Genomic Data Commons (GDC). Data assessed 31st October 2022.

In this article, we presented genomic studies that advanced our understanding of breast cancer subtypes and additional genetic studies that uncovered further molecular subtypes. In highlighting intertumoral heterogeneity, we have also discussed the importance of the tumor microenvironment in shaping the intratumoral landscape, and its impact on prognosis. Although high throughput technologies have widened our knowledge of the genomic landscape of breast cancer, most of this has been done on early-stage disease (Vasan et al., 2014; Basho et al., 2016; Bertucci et al., 2019). More advanced-stage disease may not reflect or rely on the initial driver mutations, with passenger mutations taking on a more aggressive role (Gerlinger et al., 2014). Late-stage disease may reflect the acquisition of more defined roles by selected tumor clones as a function of intratumoral heterogeneity. This could include a subpopulation with intrinsic pro-tumorigenic characteristics that persists, compared to a subpopulation that is selected for based on characteristics associated with enhanced dispersal and invasion, for example,. Nevertheless, it has been suggested that the genomic profile of the primary tumor may reflect somatic mutations in CTCs and may hold weight in distinguishing what would be deemed clonally unrelated or independent primary tumors from relapses (Yates et al., 2017). However, primary tumors, commonly used to determine the molecular characteristics of a tumor, often contain a lower proportion of metastatic clones, thus underrepresenting the metastatic potential of the tumor (Haffner et al., 2013; Bousquet et al., 2015). Moreover, while ER+ and triple-negative primary tumors can be differentiated by distinct combinations of driver mutations or copy number alterations, the genomic profile overlaps more as tumors acquire more aggressive traits (Yates et al., 2017). For example, data obtained from NCI Genomic Data Commons (GDC) showed a large disparity between the number of primary cases versus metastatic ones (Grossman et al., 2016) (Figure 3). Analysis of this data shows that there are mutations unique to metastasis disease which may play a role in its pathogenesis.

Typically, clinical trials of repurposed or novel treatments are conducted on patients with more advanced tumors that may show a variable mutational profile and definitive alteration in the TME. In metastatic tumors, extinction events caused by therapy are unlikely and reflect the acquisition of selective advantages by tumor cells to persist, disperse and colonize. The resulting tumor clones would hold spatial and temporal diversity that is only further impacted by ecological interactions with diverse cell types in the TME. There is a need to conduct more studies that seek to understand metastasis, and the role of the TME therein, in breast cancer patients as it is well-known that metastatic patients have a poorer diagnosis even with treatment.

Analyzing and interpreting data from mutational analysis tests has several challenges. For example, applying the principle of significantly mutated gene (SMG) test in analyzing the cancer genome has not been effective in identifying novel markers and possible drug targets for luminal tumors due to the underestimation of the role played by rare mutations which may act as drivers for tumor progression in certain cases such as ER-tumors (Ellis, 2013). Considering the high prevalence of breast cancer, rare mutations in breast cancer may still translate to many clinical cases being diagnosed. However, cognizance must be given that routine microarray analysis may not be able to detect splice variants, for example, the ERα ERΔE7 variant that while dysfunctional, provides a false positive for ER status and thus results in poor response to hormone therapy (Groenendijk et al., 2013; Grant et al., 2019). Another significant issue in identifying mutations in key breast cancer-associated genes is the discovery of variants whose effects are unknown. Known as variants of unknown significance (VUS), they can have implications on decision-making regarding the treatment and management of the disease (Easton et al., 2015). These variants reiterate the need for population-based studies to help clarify if these variants could be ubiquitous or related to the disease. Furthermore, functional studies are required to delineate the effect of the variants. It is also important to note the disparity in the available genomic data across different ethnicities and populations. This presents a critical challenge as most of the tools being developed for detecting and interpreting genetic information may be biased towards a specific group. It is pertinent that more studies are conducted in underrepresented populations to identify possible novel variants and their clinical relevance.

In considering these novel variants, the impact thereof with cells of the TME in driving spatiotemporally defined subclones and thus intratumoral heterogeneity must be considered. We suggest that such clones follow branched evolutionary trajectories that permit rapid selection for adaptive features that are ultimately associated with later stages of the disease. Advances in technologies including next-generation sequencing show considerable utility in unpacking not only genome-wide mutations but transcriptomic profiles that highlight real-time alterations that drive tumor progression. Nevertheless, there is still a challenge in identifying suitable patients and accessing these targeted therapies due to the associated cost of sequencing, limited specimens not fixed in formalin, consolidation of records and variability in laboratory processing. Intratumoral heterogeneity additionally impacts the utility of targeted therapy. This is highlighted in metastatic breast cancer where the mutation frequency is lower than 10% in most genes, thus providing challenging conditions regarding targeted therapy (Yates et al., 2017). Such data directs us to investigate the role of the TME in providing selective pressures for adaptation that may explain poor response to therapies and the acquisition of resistance. This concept can be further expanded, considering the TME as a fitness landscape (Phan et al., 2021) with features of microniches that drive the selection of plastic phenotypes for tumor progression. As proposed by Pienta and others, we suggest that tumor therapies consider multimodal mechanisms of drug action, or sequential regimens that limit tumor evolvability in early stages of the disease, exploiting the concept of inducing an extinction event and preventing the reconstitution of the tumor (Pienta et al., 2020). Combination treatment targeting distinct oncogenic pathways may provide a route to increasing drug efficacy and overall survival; however, there remain limitations of dose tolerability, serious adverse effects and toxicity that require investigation. Moreover, we suggest that not only should tumor cells be targeted by inhibiting pathways involved in proliferation, survival and invasion, but that the TME should be targeted by considering the re-activation of the immune system and other cells of the TME to support anti-tumor functions. We further propose that an understanding of the underlying evolutionary principles in shaping the ecology of the TME, driving fitness advantages in tumor cells and generating heterogenous microniches, will better inform precision-based approaches for breast cancer management and treatment.
